# Nothing new under the sun, or the moon, or both

**DOI:** 10.3389/fnhum.2015.00588

**Published:** 2015-11-03

**Authors:** Luca L. Bonatti, Paolo Cherubini, Carlo Reverberi

**Affiliations:** ^1^Institución Catalana de Investigación y Estudios Avanzados and Universitat Pompeu FabraBarcelona, Spain; ^2^Department of Psychology, University of Milano-BicoccaMilan, Italy; ^3^NeuroMi - Milan Center for NeuroscienceMilan, Italy

**Keywords:** reasoning, deduction, probability, dual system theory, mental models, mental logic, neuroimaging

The investigation of the mechanisms and principles of human reasoning is as ancient as the history of philosophy. It has always been clear that there is something special that allows humans, to a greater degree than other animals, to think about future states, make plans, have rational discussions, handle complex social situations, and invent marvelous things such as science. What this “something” was, however, has remained buried in mystery, and it still partially is. At the same time, demonstrations of human rationality have always been countered by staggering examples of bad reasoning, in history, in psychology, and, as many people (not us) will admit, in personal experience. The camp of psychologists and philosophers has thus been divided among those who were more impressed by the successes of humans against nature (Aristotle, Bacon, Descartes, Kant, or closer to us, the neopositivists; in psychology, Johnson-Laird, Holyoak, Newell and Simon, the Mental Logic camp) and those who were more impressed by their miserable failures (Bacon, Schoepnhauer, Kierkegaard, the nichilists, or the deconstructivists; in psychology, Tversky, Kahnemann, Evans, etc.). The latter group has argued that developing a theory of rational/logical reasoning is doomed because there is no object to study. The former group has tried to explain the (admittedly limited) rationality of the mind by developing theories of the mental representations and processes involved in deductive, causal, or probabilistic reasoning (O'Brien, 1995; Braine and O'Brien, 1998; Goldvarg and Johnson-Laird, 2001; Johnson-Laird, 2010): call this approach the Not-So-New Paradigm.

Recently, a way to reconcile the angelic and the demoniac aspects of human reasoning has taken the form of a single theory, the Dual System theory. As its name says, it replaces two alternative theories with one single theory which postulates two alternative subsystems. One may get the impression that the Dual System theory amounts to a mere reshuffling of the problems it was supposed to address, however, some of its claims may make it more than a simple trick of cards. The theory holds that one of the two systems is evolutionarily ancient, implicit, fast, mostly geared to track statistical regularities, whereas the second system is explicit, slow, effortful, error-prone, evolutionarily more recent, and perform abstract and logical reasoning. It is the characteristics of this second system that explain human errors with logical or complex probabilistic problems. Merge Bayesianism to this theory and you get what Oaksford calls the “New Paradigm,” which, he writes, is “based on Bayesian probability and dual processes” (Oaksford, 2015). Not only does the New Paradigm offer a novel theoretical framework to advance our knowledge of human reasoning, but it also offer “an alternative theoretical framework to those typically assumed in imaging research on deductive reasoning.”

We cannot feel the same enthusiasm. First, it seems to us that explaining human reasoning by constraining it within the dual system theory is overly optimistic. Even within the narrow realm of deductive reasoning, many systems are likely involved. Certainly beyond deduction a whole constellation of inferential systems exist, and the interaction between them is neither simple nor predictable along the very rough boundaries provided by the dual system theory. Infants seem to be able to draw correct probabilistic inferences, both before and after being able to verbalize their reasoning (Téglás et al., 2007, 2011, 2015), but it is not clear if these abilities are implicit or explicit. So, does probabilistic reasoning belong to System 1 or 2?

There is also strong evidence that rational problem solving is deeply entrenched in the human mind at its earliest stages. Infants understand goals and the optimality of actions in a variety of situations difficult to capture by the postulation of a single, non-rational, system (Gergely et al., 1995, 2002; Csibra, 2008; Csibra and Gergely, 2009; Southgate and Csibra, 2009); they explore unknown situations making very specific hypotheses and testing them (Gweon and Schulz, 2011; Stahl and Feigenson, 2015); and they know how to interpret simple probabilistic situations and how event probabilities change in many different contexts (Téglás et al., 2011, 2015). What system do these abilities belong to, and, is it useful to even ask this question? With the little we know about basic reasoning abilities and their development, it is hard to see how jumping from paradigm to paradigm can help in developing the necessary knowledge. Finally, as Oaksford himself recalls, the Dual System theory cuts the pie in the wrong way. For example, it is an assumption of the theory that errors in deductive reasoning depend on it being a System-2-kind of phenomenon. However, we now know that an important part of deduction is implicit (De Neys and Schaeken, 2007; De Neys, 2012; Reverberi et al., 2012b), and that many easy deductive inferences are fast, spontaneous, and make no use of working memory to hold intermediate conclusions (Braine and O'Brien, 1998; Johnson-Laird, 2010), something that would make them a System-1-like process. Again, does deduction belong to System 1 or System 2? We believe that the best way to address this question is to refuse to answer in terms of a theory that is too coarse to provide any substantial answer. In short, we fail to see what is new in the New Paradigm, insofar as its novelty depends on the adoption of the Dual System theory.

Second, besides the Dual Theory, the novelty of the New Paradigm entirely consists of its probabilistic claim, mostly spelled out in a Bayesian framework. We agree with Oaskford that Bayesianism has made substantial new progress in the understanding of human reasoning, although the framework is so powerful that it is difficult to find its limits (Endress, 2013). However, it is an illusion to think that such progress is reason to dismiss the very same questions with which the Not-So-New paradigm struggles. Bayesianism is a theory about how hypotheses change in the face of experience. There is no Bayesian Theory to begin with, if one does not specify the language with which the very same hypotheses whose degree of confidence should change are framed. This language is going to involve a logic, because it has to incorporate logical connectives, quantifiers, modal operators, epistemic operators, and the like—precisely the kind of objects that the Not-So-New paradigm aims at studying (Tenenbaum et al., 2006; Stuhlmüller and Goodman, 2014). In short, the New Paradigm holds that most knowledge is probabilistic, but that probabilstic knowledge must lie on a bed of logical representations and of logical inference. So if you want a new paradigm, you'd better develop the Not-so-New paradigm along.

Given all the above, understanding how the human brain implements the elementary building blocks of human deductive competence is a fundamental goal. Neuroimaging can and has been used to inform/constrain psychological theories of deduction (see also Henson, 2005; Heit, 2015). However, Oaksford argues that many studies mistakenly understood as imaging deduction concern “elaborative, defeasible, and probabilistic reasoning”, thus suggesting that imaging data do not support the existence of deduction mechanisms. We believe these criticisms underestimate the methodological and experimental progress that the neuropsychology of reasoning, inspired by the Not-So-New paradigm, has made in these last 15 years.

First, many studies already factor in the methodological criticisms raised by Oaksford. For example, it has been pointed out that specific task demands may greatly modify how participants solve deductive problems, e.g., by using analytic or heuristic processing (Reverberi et al., 2009a). The importance of choosing an adequate baseline has also been emphasized (Monti et al., 2007; Reverberi et al., 2007), or appropriate behavioral indices (Rotello and Heit, 2014). Also, recent studies consider between subject variability and try to identify fine-grained functional specializations within the network involved in deduction (e.g., Reverberi et al., 2010).

Second, recent convergent findings “deductive tasks” can be naturally interpreted within the framework of the Not-So-New paradigm:

1. The left ventro-lateral prefrontal cortex (left VLPFC, Brodmann Area 47/10) is active when participants are either evaluating or generating new deductive conclusions, both when the problems are abstract, and when they contain thematic information (Monti et al., 2007, 2009; Reverberi et al., 2010; Prado et al., 2014). Furthermore, activity in the left VLPFC predicts whether individuals tend to generate valid answers to deductive problems (Reverberi et al., 2012a), and is modulated in tasks requiring to evaluate compatibility of simple propositional sentences with evidence (Baggio et al., 2015).
2. The posterior portion of the left inferior frontal gyrus (left IFG, mostly BA44/45) is involved in inference making (Baggio et al., 2015; see also Goel et al., 2000; Reverberi et al., 2007, 2010; Prado et al., 2011), and recent studies trace its contribution to logical forms. Specifically, activity in left IFG predicts whether or not participants extract and use the formal structure of deductive problems for generating a conclusion. Importantly left IFG activation does not predict whether the generated conclusion will be valid or not, suggesting that its role is less the active process of drawing a conclusion than that of representing the logical form (Reverberi et al., 2012a). Converging evidence suggests that left IFG is devoted to computing hierarchies and relations among trees (Pallier et al., 2011). Again, these results account for individual differences, and suggest the presence of a cascade of mental representations well predicted by the Not-So-New paradigm.
3. Functional dissociations have been reported between deductive tasks of different types, such as relative and propositional reasoning (Prado et al., 2010), or conditional and categorical reasoning (Reverberi et al., 2010). Furthermore, some part of the reasoning network (e.g., VLPFC) have been shown to dissociate “logic” from “linguistic arguments” (Monti et al., 2009).

These results prompted revisions of too-coarse-grained versions of theories of deductive reasoning (Monti et al., 2009; Reverberi et al., 2009b; Prado et al., 2010), but they also confirmed a neuroimaging approach inspired by main tenets of the Not-So-New paradigms: content can be separated from form, logical form from inference; strict predictive relations exist between patterns of brain activities and individual differences in participants' solution strategies. By contrast, we find the New Paradigm in this context predictively sterile: we fail to see what novel or different predictions it would bring about.

Perhaps future progress can be made by changing paradigm. Certainly, we agree with Oaksford and others (e.g., Heit, 2015) that the field would benefit from computational modeling, and further theoretical development. But we believe there is still much juice to be gained by squeezing the Not-So-New paradigm. The perspective of progress it offers should not be overlooked.

## Conflict of interest statement

The authors declare that the research was conducted in the absence of any commercial or financial relationships that could be construed as a potential conflict of interest.
